# Supplementary material for: Training Generalized Segmentation Networks with Real and Synthetic Cryo-ET data
Source: bioRxiv. 2025 Feb 5:2025.01.31.635598. Preprint. [Version 3] doi: 10.1101/2025.01.31.635598 (PMC11838407; doi:10.1101/2025.01.31.635598)
Supplement: 1 [file NIHPP2025.01.31.635598v3-supplement-1.pdf]

bioRxiv preprint doi: https://doi.org/10.1101/2025.01.31.635598; this version posted February 5, 2025. The copyright holder for this preprint (which was not certified by peer review) is the author/funder, who has granted bioRxiv a license to display the preprint in perpetuity. It is made available under aCC-BY 4.0 International license.

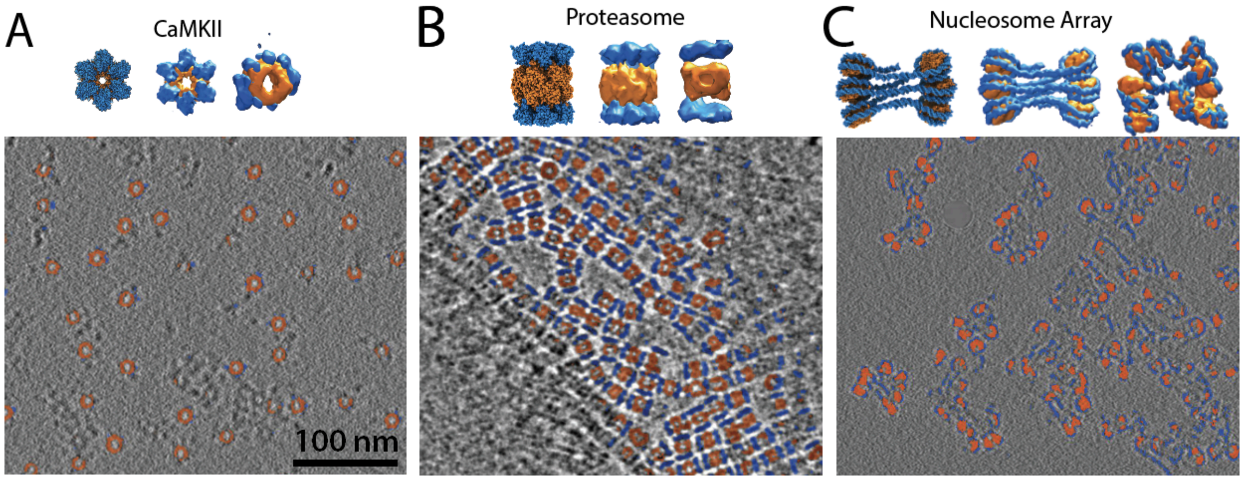

Figure S1: *Sub-molecular segmentation using synthetic data*

(A) The top panel contains a space-filling atomic model of CaMKII (PDB 3SOA), that model rendered as a colorized density map, and a 3D rendering of a segmented molecule from the corresponding tomogram in the lower panel (EMD-41263) showing segmentation of the core (orange) and catalytic domains (blue). (B) Similar panels showing the approaches effectiveness in segmenting the outer and inner rings of purified proteasomes in EMD-7152 (PDB 5FMG). (C) Similar panels showing the approaches effectiveness in segmenting DNA and histones within purified nucleosome arrays (PDB 6HKT).

bioRxiv preprint doi: https://doi.org/10.1101/2025.01.31.635598; this version posted February 5, 2025. The copyright holder for this preprint (which was not certified by peer review) is the author/funder, who has granted bioRxiv a license to display the preprint in perpetuity. It is made available under aCC-BY 4.0 International license.

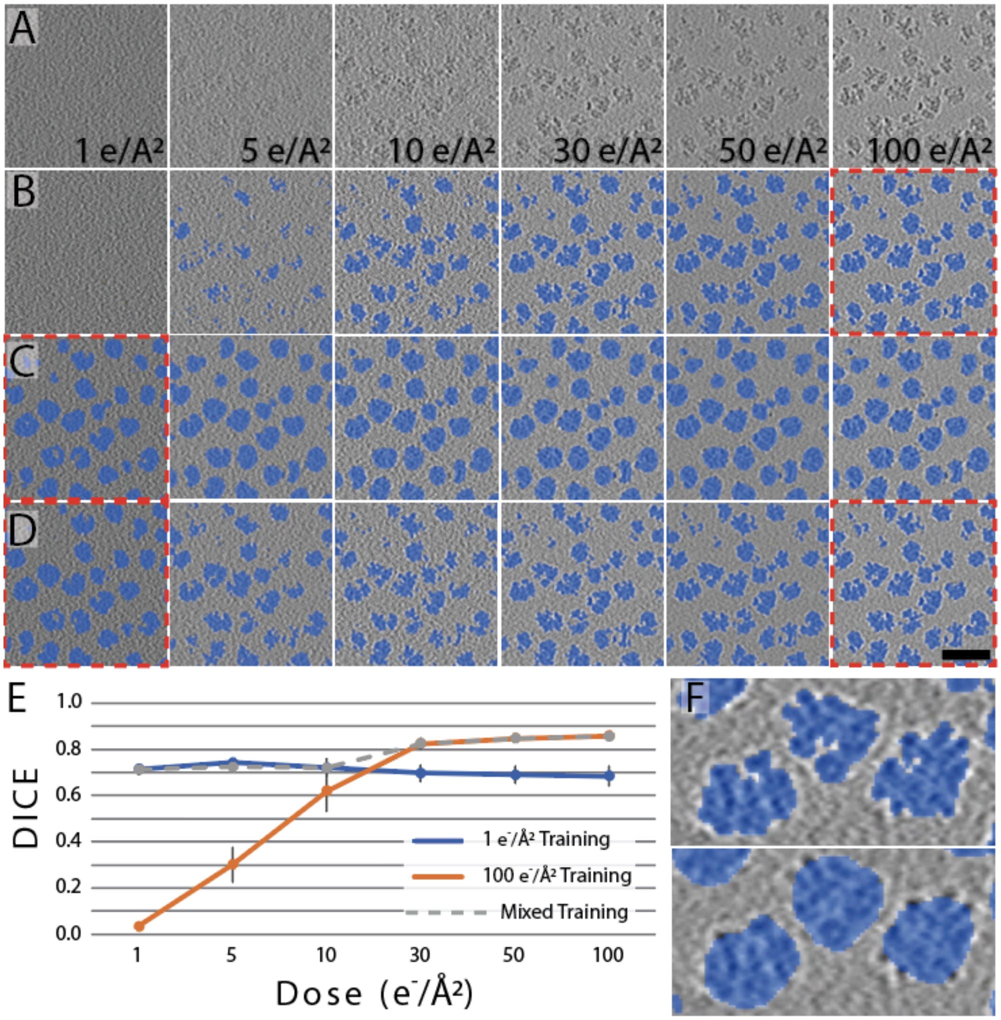

Figure S2: *The effect of electron dose on training segmentation networks.*

(A) A series of synthetic tomograms generated from the same model (randomly oriented ribosomes), but with different electron dose (-1). (B) Ribosomal segmentations (blue) generated from a single network trained on the highest-dose dataset (red-dashed box). (C) Ribosomal segmentations generated from a single network trained on the lowest-dose dataset (red-dashed box). (D) Ribosomal segmentations generated from a single network trained on both the highest- and lowest-dose datasets (red-dashed boxes). (E) Line graph of the DICE scores for each segmentation compared in terms of electron dose. (F) Close-up views of segmentations from 100 $e^{-}/Å^{2}$ simulations in (C) and (D), Top and Bottom, respectively.

bioRxiv preprint doi: https://doi.org/10.1101/2025.01.31.635598; this version posted February 5, 2025. The copyright holder for this preprint (which was not certified by peer review) is the author/funder, who has granted bioRxiv a license to display the preprint in perpetuity. It is made available under aCC-BY 4.0 International license.

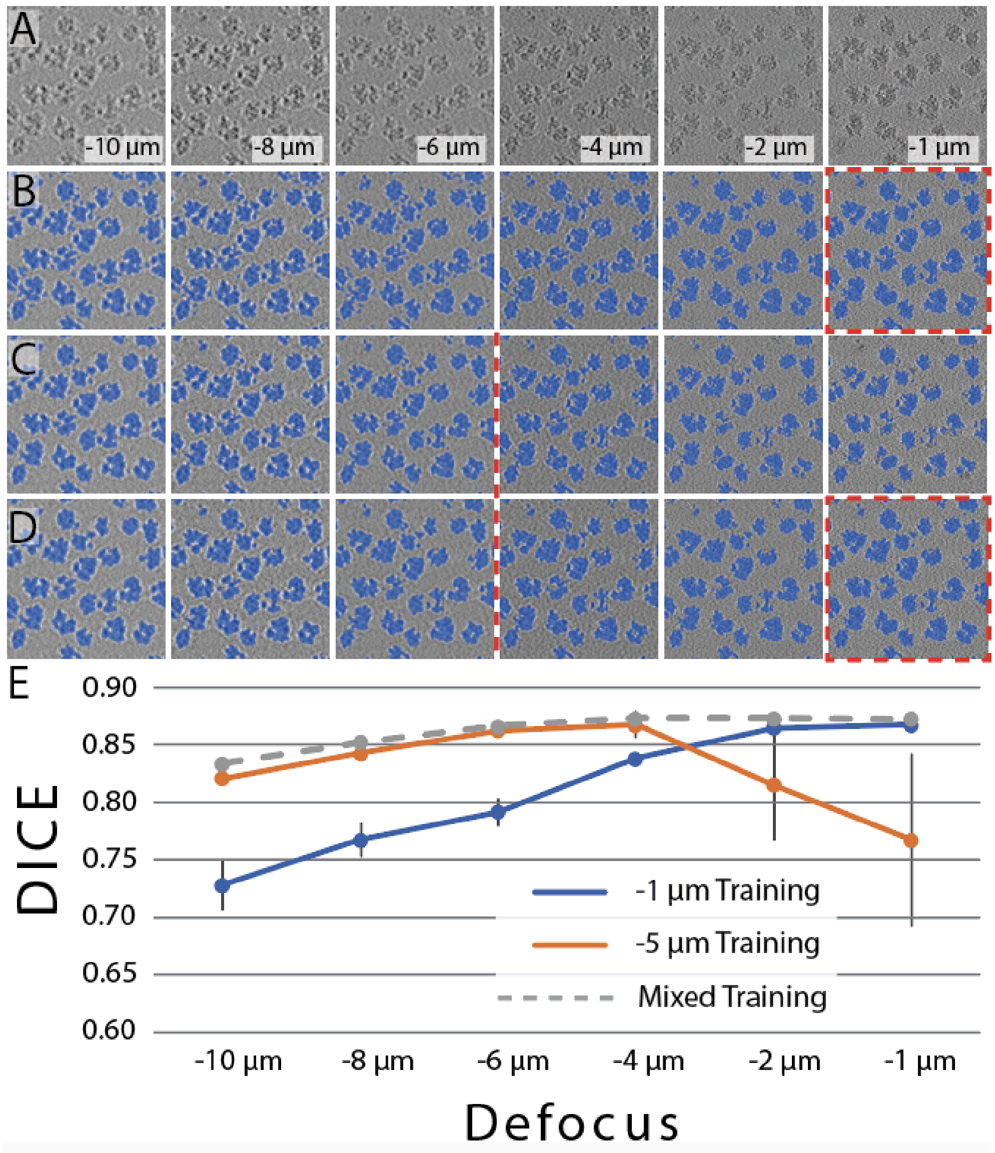

Figure S3: *The effect of defocus on training segmentation networks.*

(A) A series of synthetic tomograms generated from the same model (randomly oriented ribosomes), but with different defocus values. (B) Ribosomal segmentations (blue) generated from a single network trained on the -1 µM defocus dataset (red-dashed box). (C) Ribosomal segmentations generated from a single network trained on a -5 µM defocus dataset (red-dashed line). (D) Ribosomal segmentations generated from a single network trained on both the -1 and -5 µM defocus datasets (red-dashed line and box). (E) Line graph of the DICE scores for each segmentation compared in terms of image defocus.

bioRxiv preprint doi: https://doi.org/10.1101/2025.01.31.635598; this version posted February 5, 2025. The copyright holder for this preprint (which was not certified by peer review) is the author/funder, who has granted bioRxiv a license to display the preprint in perpetuity. It is made available under aCC-BY 4.0 International license.

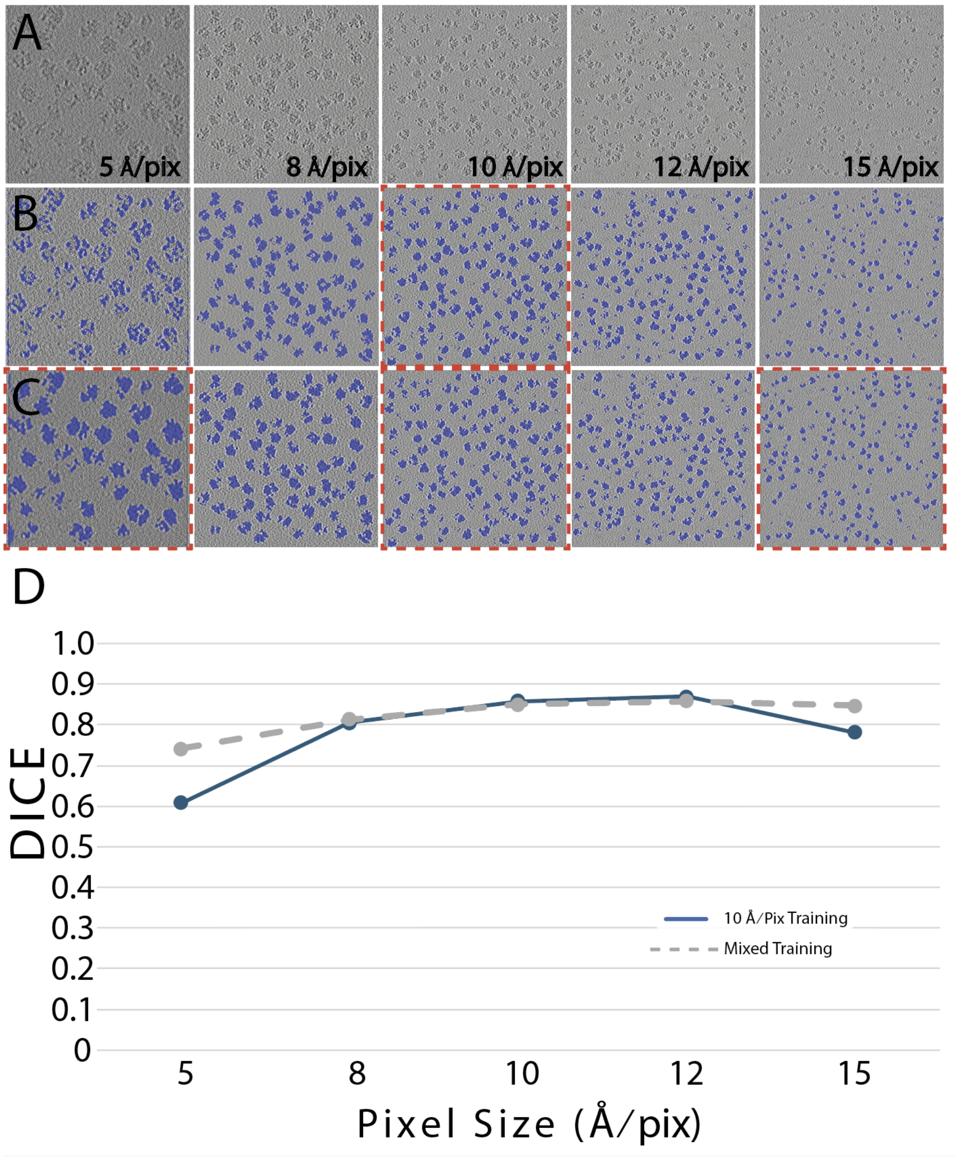

Figure S4: *The effect of Pixel Size on training segmentation networks.*

(A) A series of synthetic tomograms generated from models of randomly oriented ribosomes along a gradient of pixel sizes (5-15 ang/pix). (B) Ribosomal segmentations (blue) generated from a single network trained at 10 ang/pix (red-dashed box). (C) Ribosomal segmentations generated from a single network trained at 5, 10, and 15 ang/pix (red-dashed boxes). (D) Line graph of the DICE scores for each segmentation compared in terms of pixel size.

bioRxiv preprint doi: https://doi.org/10.1101/2025.01.31.635598; this version posted February 5, 2025. The copyright holder for this preprint (which was not certified by peer review) is the author/funder, who has granted bioRxiv a license to display the preprint in perpetuity. It is made available under aCC-BY 4.0 International license.

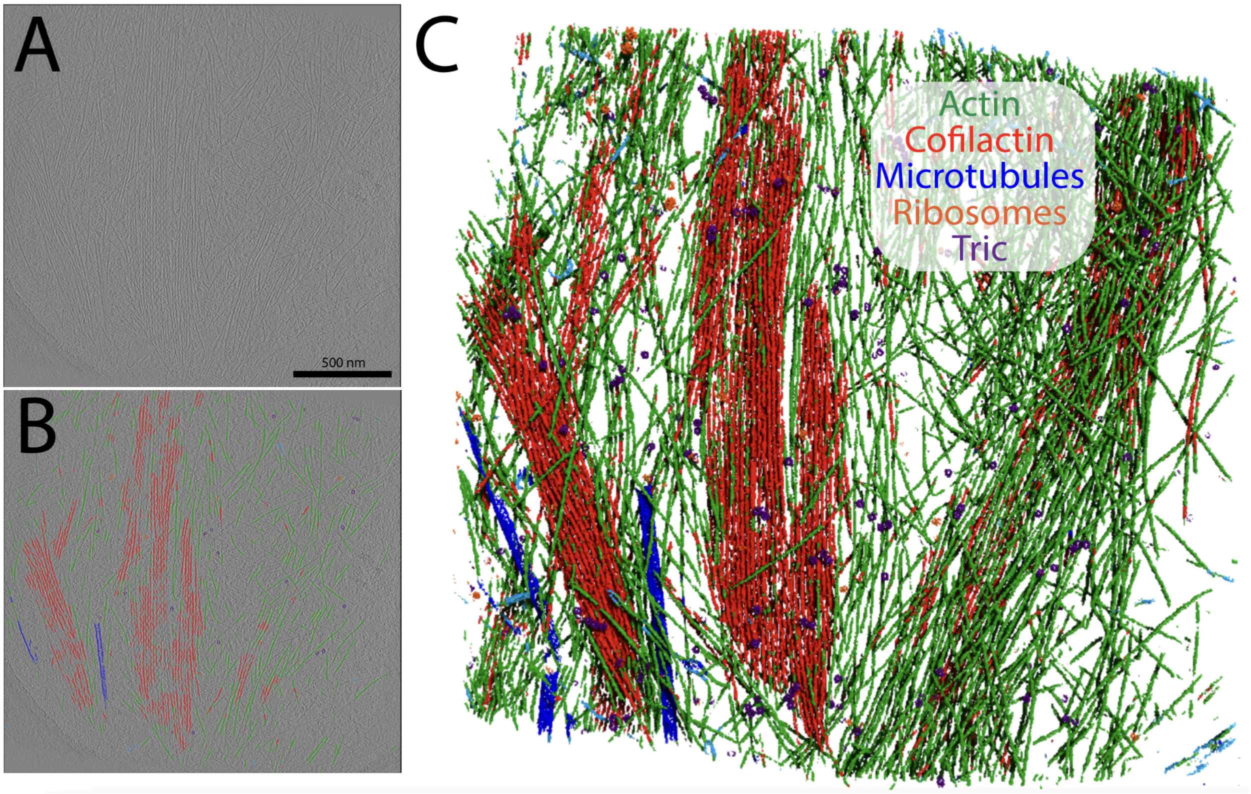

Figure S5: *Co-trained networks easily differentiate between actin and cofilactin in situ*.

(A) Slice from a cryotomogram taken within the transition zone of a neuronal growth cone, and (B) its segmentation by a co-trained UNet. (C) High-resolution 3D segmentation of the tomogram in (A), where bare actin and cofilactin are distinguished either individually, as bundles, or mixed bundles.

bioRxiv preprint doi: https://doi.org/10.1101/2025.01.31.635598; this version posted February 5, 2025. The copyright holder for this preprint (which was not certified by peer review) is the author/funder, who has granted bioRxiv a license to display the preprint in perpetuity. It is made available under aCC-BY 4.0 International license.

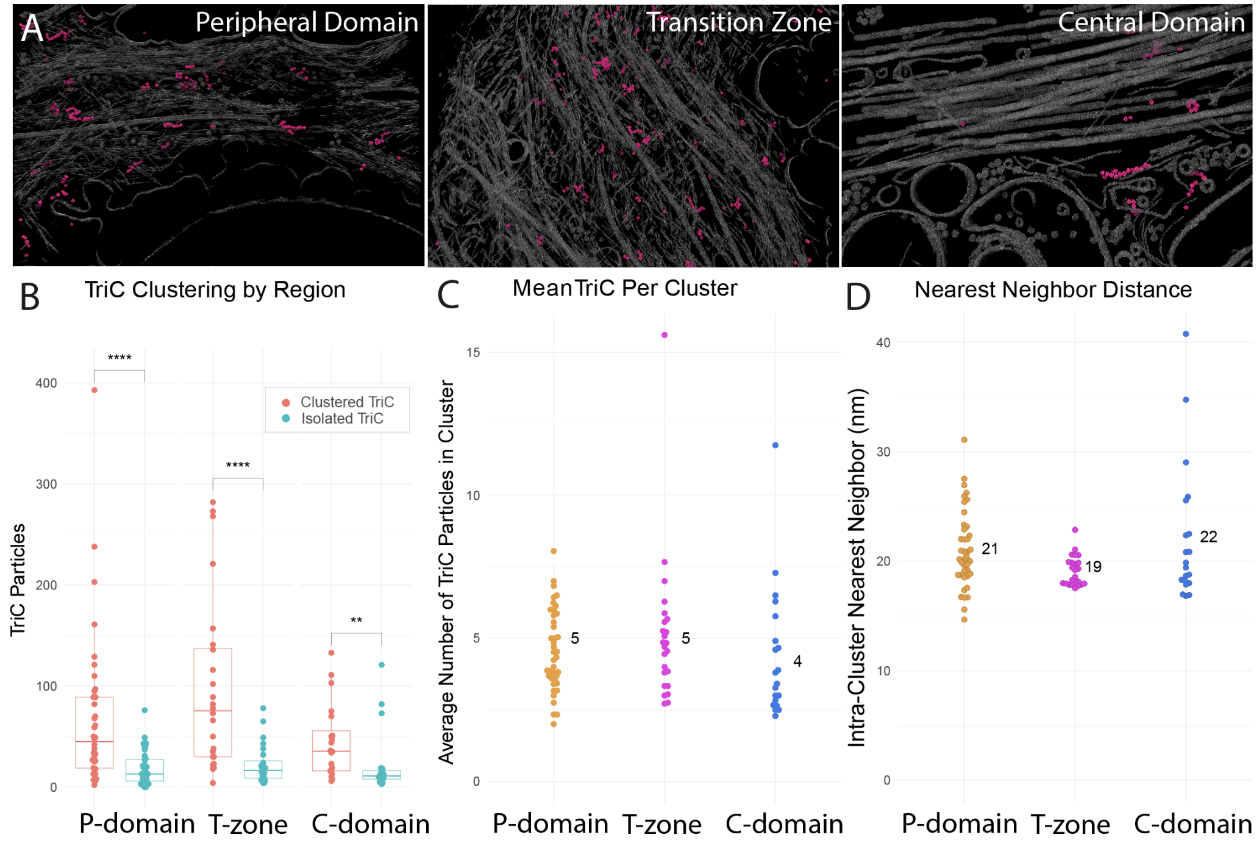

Figure S6: *TriC clustering in growth cones*

(A) Representative segmentations of TriC clusters (pink) in tomograms from each domain of the growth cone. (B) Distribution of isolated and clustered TriC across domains. (C) Mean number of TriC particles in each cluster across domains. (D) Measured intra-cluster nearest neighbor distance for TriC.

bioRxiv preprint doi: https://doi.org/10.1101/2025.01.31.635598; this version posted February 5, 2025. The copyright holder for this preprint (which was not certified by peer review) is the author/funder, who has granted bioRxiv a license to display the preprint in perpetuity. It is made available under aCC-BY 4.0 International license.

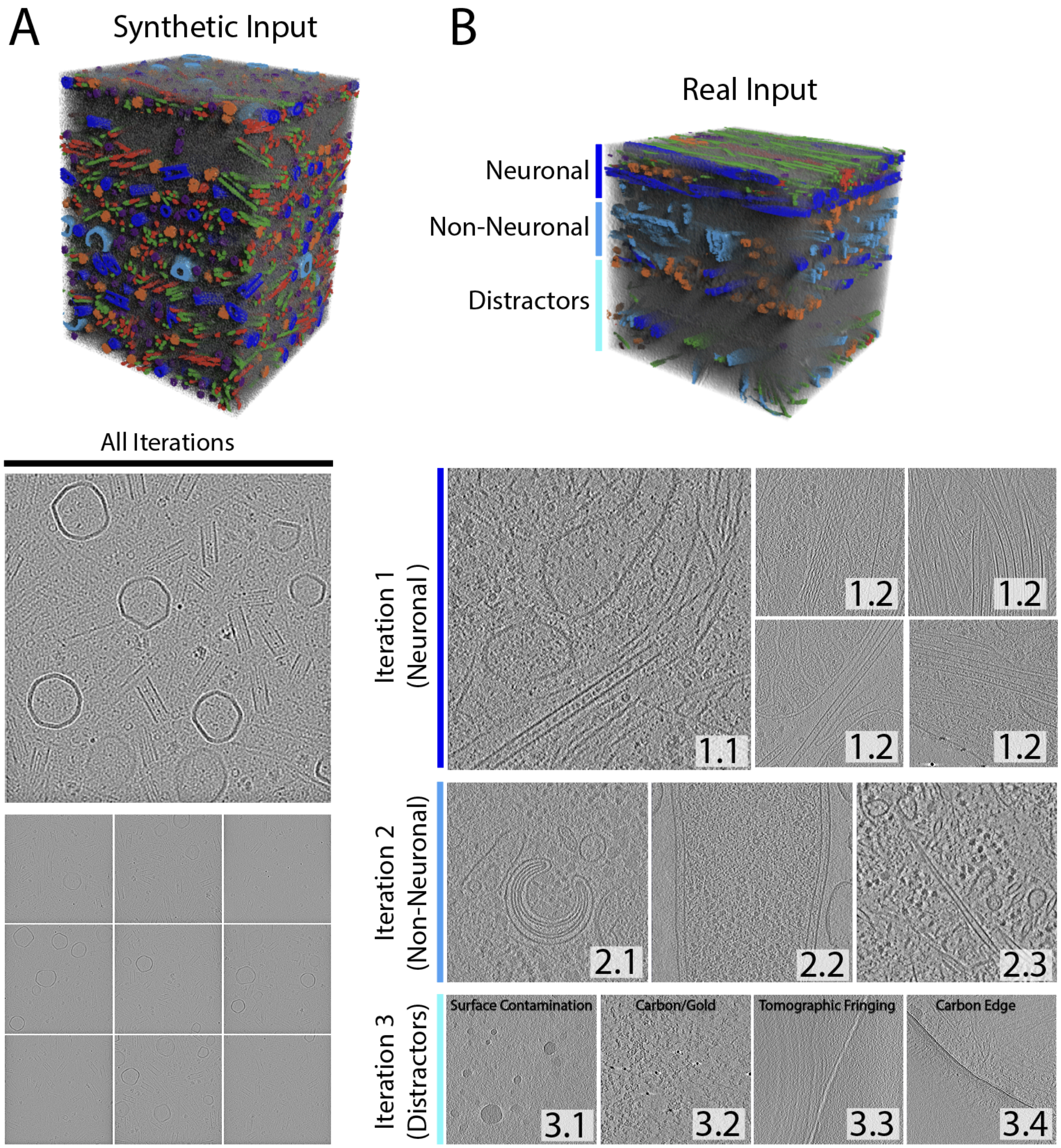

Figure S7: *Training inputs for the development of NeuroSeg*

(A) Block of concatenated synthetic tomographic data. All synthetic tomograms are 400x400x50 voxels and generated programmatically by CTS to vary randomly within a set range of both modeling and simulation parameters. In the lower panel, individual synthetic tomograms are displayed. They contain the primary targets (membrane, microtubules, actin, cofilactin, ribosomes and TriC) as well as randomized sets of molecules from a pool of distractor PDBs. (B) Block of concatenated real tomographic

bioRxiv preprint doi: https://doi.org/10.1101/2025.01.31.635598; this version posted February 5, 2025. The copyright holder for this preprint (which was not certified by peer review) is the author/funder, who has granted bioRxiv a license to display the preprint in perpetuity. It is made available under aCC-BY 4.0 International license.

data. All real tomograms are 400x400xZ voxels, where Z varies between 10 and 50. During each iteration of the network, the synthetic data was used as a base for co-training with the real inputs. For each iteration, segmentations based on the previous round of training were used as a starting point for cropping and class remapping, as well as repair of false positives/negatives. Hand-corrected patches of data were then fed back into the next iteration of training.

bioRxiv preprint doi: https://doi.org/10.1101/2025.01.31.635598; this version posted February 5, 2025. The copyright holder for this preprint (which was not certified by peer review) is the author/funder, who has granted bioRxiv a license to display the preprint in perpetuity. It is made available under aCC-BY 4.0 International license.

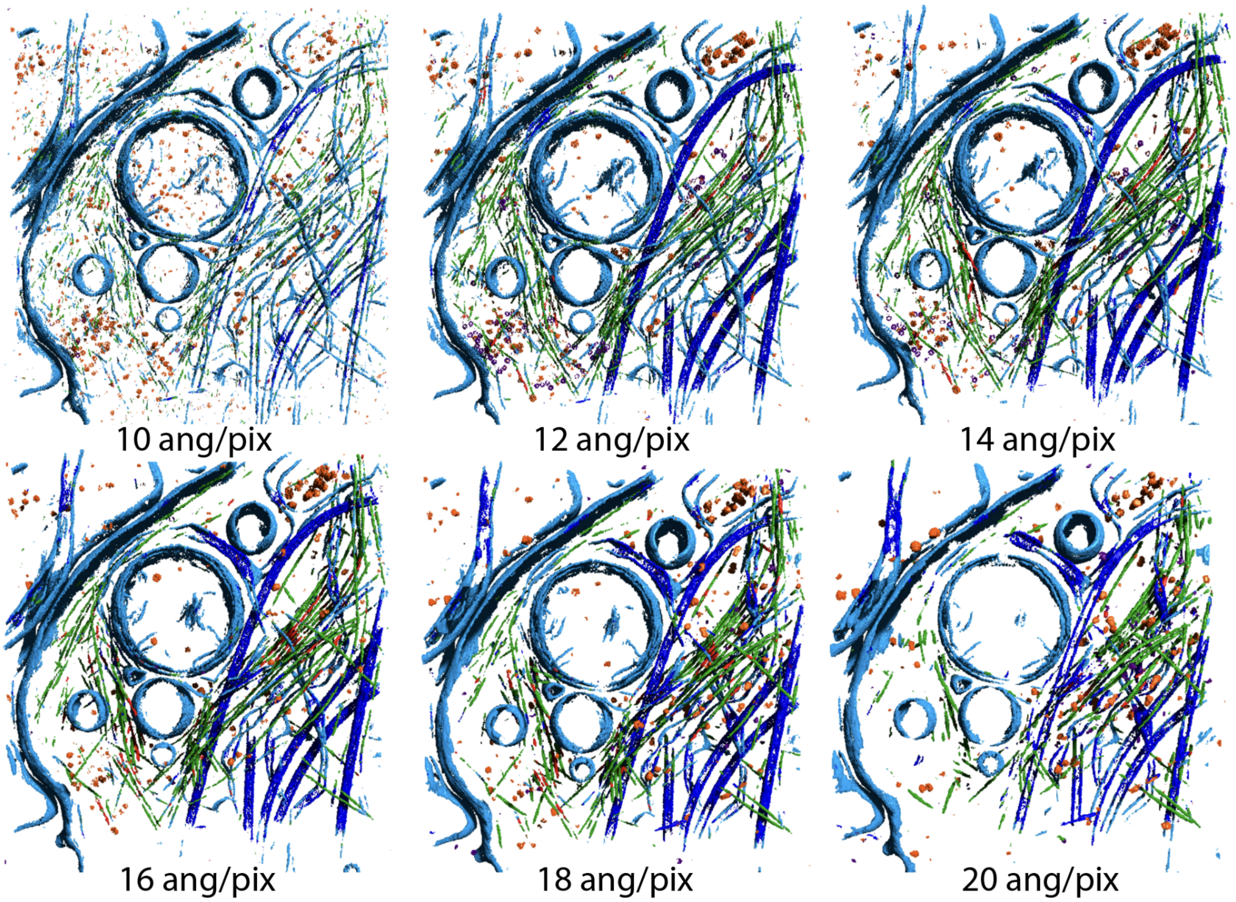

Figure S8: *NeuralSeg works across a range of pixel sizes*

At the top left is the segmentation of the original tomogram reconstructed at 10 angstrom/pixel, followed by a series of segmentations of the same tomogram after rescaling in 20% increments to 20 ang/pix.

bioRxiv preprint doi: https://doi.org/10.1101/2025.01.31.635598; this version posted February 5, 2025. The copyright holder for this preprint (which was not certified by peer review) is the author/funder, who has granted bioRxiv a license to display the preprint in perpetuity. It is made available under aCC-BY 4.0 International license.

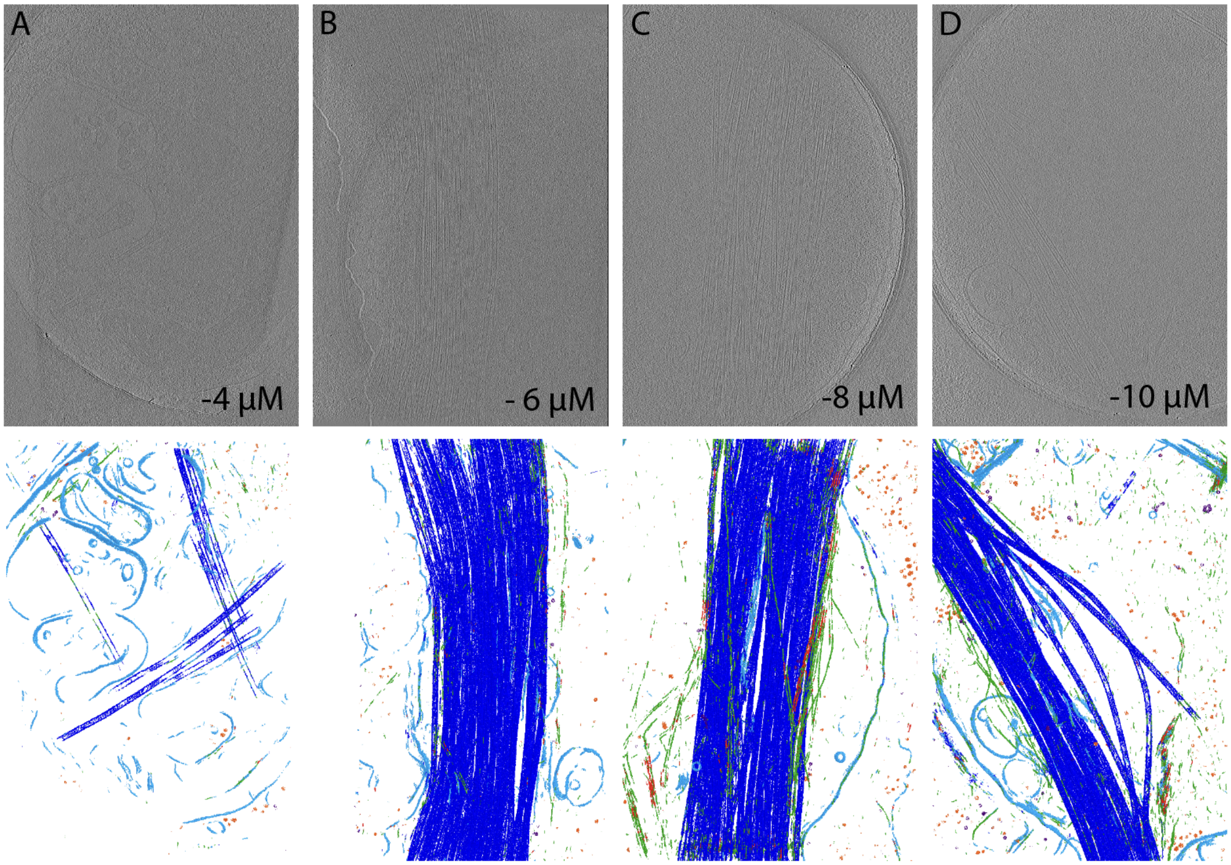

Figure S9: *NeuralSeg works across the range of defocus from -4 to -10 μM*

Top panels are slices through neuronal tomograms collected at different defoci, from -4 to -10 μM, and below are their corresponding segmentations by NeuralSeg.

bioRxiv preprint doi: https://doi.org/10.1101/2025.01.31.635598; this version posted February 5, 2025. The copyright holder for this preprint (which was not certified by peer review) is the author/funder, who has granted bioRxiv a license to display the preprint in perpetuity. It is made available under aCC-BY 4.0 International license.

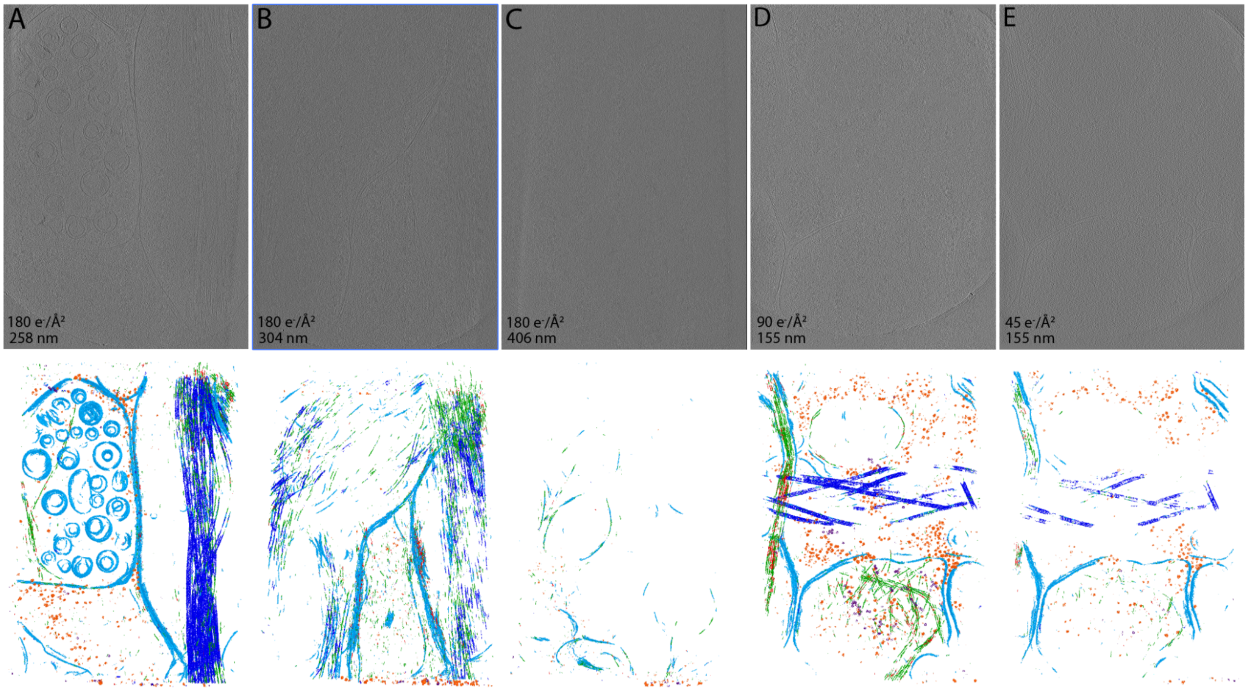

Figure S10: *Tomogram contrast resulting from thickness and electron dose affects NeuralSeg's performance*

Top panels are slices through six tomograms and their corresponding segmentations by NeuralSeg on the bottom. (A) through (C) are tomograms collected at the same electron dose of 180 $e^-/Å^2$ but have increasing thickness from 258 nm to 406 nm. (D) and (E) are tomograms of the same position, and therefore the same thickness of 155 nm, but at different electron doses, 90 $e^-/Å^2$ and 45 $e^-/Å^2$.

bioRxiv preprint doi: https://doi.org/10.1101/2025.01.31.635598; this version posted February 5, 2025. The copyright holder for this preprint (which was not certified by peer review) is the author/funder, who has granted bioRxiv a license to display the preprint in perpetuity. It is made available under aCC-BY 4.0 International license.

Protein	PDB(s)	Modifications
Actin	6t1y	Elongated helically to 39 subunits.
Cofilactin	3j0s	Elongated helically to 33 subunits.
Microtubules	6o2t	Elongated helically by 2x, filled with 3 MIPS.
CaMKII	3soa, 5u6y	Split into catalytic and association domains.
Proteasome	5fmg	Split into alpha and beta component submodels.
Chromatin Array	6hkt	Spit into DNA and histones
Ribosome	6ks8	
TriC	4ujd	
Distractors		
Small (<200 kDa)	2q0u, 1sjj, 1qtx, 2cg9, 4uic, 5csa, 6vgr, 7e6g, 7wbt, 1exr	
Large (>200 kDa)	1qvr, 2dfs, 3soa, 5fmg, 5o32, 6igc, 6ksp, 6u8q, 7bkc, 7nhs	

Table S1: *PDBs and their modifications for simulation*

bioRxiv preprint doi: https://doi.org/10.1101/2025.01.31.635598; this version posted February 5, 2025. The copyright holder for this preprint (which was not certified by peer review) is the author/funder, who has granted bioRxiv a license to display the preprint in perpetuity. It is made available under aCC-BY 4.0 International license.

CTS Overview

Modeling Approach

CTS models are essentially Coulomb potential maps based on atomic structure files (.pdb and .cif) with limited support for models based on density maps. CTS follows the weak phase object approximation, which is sufficient for most cryoEM specimens. Models are generated primarily using brute force, with individual structures tested at potential insertion sites several times, repeated over many iterations. Use of efficient code allows CTS to generate models quickly, with small models (~400x400x100 px) taking only a few minutes to create depending on density. CTS has separate functions for generating full atomic models and coarse voxelwise models depending on computational resources, resolution, and use case.

Modeling steps

A CTS model starts with any of the standard features opted into: a carbon support film, vesicle membranes, and boundary constraints. Input structures are then added in a series of layers set by the user, allowing precise control over relative abundance and occupancy of different inputs. Next, gold fiducial beads can be added, and the model is embedded in vitreous ice. Finally, an atlas is generated with each voxel labeled with the class of its main component alongside a simulation run, matched to its final size.

Modeling features

Clustering: structures can be placed as local clusters isotropically, or in packed bundles to model filaments like actin.
Complexes: structures can be subdivided, with each subdivision labeled separately in the output atlas. This allows subdomains of very large proteins or different proteins in a complex to be distinguished. A variant 'assembly' allows randomized occupancy of subdivisions for easier implementation of nonuniform structures.
Membrane embedding: structures can be placed in the membrane as a transmembrane or membrane-associated protein. The OPM database provides compatible structure files, but CTS has a tutorial for adapting any structure for use in this way with UCSF Chimera.
Vesicle placement: structures can be placed exclusively inside membrane-bound vesicles or exclusively outside, not merely globally random locations.

Simulation approach

CTS also uses a coarse-grained method to generate simulations from input models, operating on bulk interactions at a pixel level rather than atomic and wave interactions. This enables fast runtimes on minimal hardware, without sacrificing utility for our deep learning purposes. CTS has a large number of input parameters whose default values

bioRxiv preprint doi: https://doi.org/10.1101/2025.01.31.635598; this version posted February 5, 2025. The copyright holder for this preprint (which was not certified by peer review) is the author/funder, who has granted bioRxiv a license to display the preprint in perpetuity. It is made available under aCC-BY 4.0 International license.

cover modern hardware but can cover a range of possibilities from standard to reasonable and even technically impossible imaging capabilities. Primary control parameters are the same as standard imaging parameters: microscope characteristics and imaging factors including tilt increment and limits, defocus, electron dose, and tilt scheme. Advanced options include control over radiation damage, deviation from target tilt angles, inelastic electron scattering, and generation of "ideal" images that lack CTF modulation and probabilistic dose sampling. CTS has two simulator functions: the first a purely volume-based implementation that uses a 2.5d approximation of defocus and CTF, and a more intensive option that requires an atomic model but implements a fully 3d CTF at the cost of runtime.

Simulation Components

Tilt Projection

For the volumetric method, CTS currently relies on the external IMOD[24] xyzproj command to create the initial model projections. Regardless of method, CTS can project tilts around either the X or Y axis and though it expects a balanced tilt series can project any arbitrary set of angles in any order. This allows single micrographs, multiframe tilts, and complex acquisition series. CTS also provides a parameter for tilt error, which introduces a scaling random variance to the true angle that is projected from the target angle.

Dose sampling

The density contribution to contrast is simulated by sampling electrons from a distribution of the tilt angle's scattering potential (Fig. 1D). For each tilt, the camera-detected dose is adjusted based on the maximal DQE of the detector as well as inelastic scattering of electrons away from the path of the detector. This dose-adjusted scattering map is used as the lambda parameter of a poisson distribution from which detected electrons are drawn. The following equation is used to determine transmitted dose:

$$Dc = e^\wedge(- T/cos(theta) * S/IMFP) * dose * DQE$$

Dc is the corrected dose transmitted, S is the scattering factor (1), IMFP is the inelastic mean free path of vitreous ice (3.8nm), T is the thickness of the sample, theta is the tilt angle, and DQE is the detector quantum efficiency of the camera.

CTF intensity modulation

Once tilt images are projected, they are modulated by a contrast transfer function (CTF) based on the pixel size and defocus, as well as microscope parameters such as the accelerating voltage and spherical aberration (Figure 1E). As mentioned previously only with the atomic simulator is CTF modulation fully 3d, while the volumetric simulator uses

bioRxiv preprint doi: https://doi.org/10.1101/2025.01.31.635598; this version posted February 5, 2025. The copyright holder for this preprint (which was not certified by peer review) is the author/funder, who has granted bioRxiv a license to display the preprint in perpetuity. It is made available under aCC-BY 4.0 International license.

a 2.5D method with the defocus approximated in a series of overlapping 2D strips parallel to the tilt axis.

The CTF in fourier space is computed according to the following functions:

1. $CTF = E * ((1 - Q)sin(eq) + Qcos(eq))$
2. $eq = pi/2 * (CS * L^3 * k^4 - 2 * Dz * L * k^2)$
3. $E = e^{-(k/(sigma*nyquist))^2}$
4. $L = H * c/sqrt(e * V * (2 * m * c^2 + e * V))$

Overall equation for the CTF profile (1), wave component equation (2), envelope function of the contrast (3), and the calculation of the relativistic electron wavelength (4).

Where L is the relativistic electron wavelength, CS is spherical aberration, K is the spatial frequency, Dz is defocus, sigma is the envelope factor (.9), and Q is the amplitude contrast factor (.07). In 4, H is the planck constant, e the electron charge, c the speed of light, m the electron mass, and V the acceleration voltage.

<u>Radiation damage</u>

Radiation damage is modeled in a very simplified fashion. Before electrons are sampled for each tilt angle, that tilt projection is corrupted by two operations scaled by the cumulative electron dose transmitted. The first operation is a smoothing step that reduces signal clarity in higher-resolution images, and the second is a layer of gaussian noise applied to the whole tilt image.

<u>Tomographic reconstruction</u>

CTS outputs are ready for immediate reconstruction without any other processing necessary. CTS performs reconstruction of the simulated tilt series (Fig. 1F) using IMOD's tilt command with the target tilt angles, followed by using the IMOD command trimvol to rotate the tomographic reconstruction to a standard orientation. The lack of CTF correction (and alignment, in the case of introduced alignment error) are deliberate, as the baseline values for each contribute more to very small scale contrast and signal, rather than large-scale errors. These correspond to higher quality data that similarly would not need further refinement.
